# Volunteer-led online group exercise for community-dwelling older people: a feasibility and acceptability study

**DOI:** 10.1186/s12877-023-04184-7

**Published:** 2023-07-28

**Authors:** S. E. R. Lim, S. J. Meredith, S. Agnew, E. Clift, K. Ibrahim, H. C. Roberts

**Affiliations:** 1 Academic Geriatric Medicine, Southampton General Hospital, Mailpoint 807, Southampton, SO16 6YD UK; 2 NIHR Applied Research Collaboration Wessex, University of Southampton, Southampton, UK; 3 NIHR Southampton Biomedical Research Centre, University of Southampton, Southampton, UK; 4 The Brendoncare Foundation, Winchester, UK; 5 Southern Health NHS Foundation Trust, Southampton, UK

**Keywords:** Volunteer, Physical activity, Online exercise, Older adults

## Abstract

**Background:**

Despite the clear benefits of physical activity in healthy ageing, engagement in regular physical activity among community-dwelling older adults remains low, with common barriers including exertional discomfort, concerns with falling, and access difficulties. The recent rise of the use of technology and the internet among older adults presents an opportunity to engage with older people online to promote increased physical activity. This study aims to determine the feasibility and acceptability of training volunteers to deliver online group exercises for older adults attending community social clubs.

**Methods:**

This was a pre-post mixed-methods study. Older adults aged ≥ 65 years attending community social clubs who provided written consent and were not actively participating in exercise classes took part in the feasibility study. Older adults, volunteers, and staff were interviewed to determine the acceptability of the intervention. The intervention was a once weekly volunteer-led online group seated strength exercises using resistance bands. The duration of the intervention was 6 months. The primary outcome measures were the feasibility of the intervention (determined by the number of volunteers recruited, trained, and retained, participant recruitment and intervention adherence) and its acceptability to key stakeholders. Secondary outcome measures included physical activity levels (Community Health Model Activities Programme for Seniors (CHAMPS) questionnaire), modified Barthel Index, Health-related quality of life (EQ-5D-5L), frailty (PRISMA-7) and sarcopenia (SARC-F), at baseline and 6 months.

**Results:**

Nineteen volunteers were recruited, 15 (78.9%) completed training and 9 (47.3%) were retained after 1 year (mean age 68 years). Thirty older adults (mean age 77 years, 27 female) participated, attending 54% (IQR 37–67) of exercise sessions. Participants had no significant changes in secondary outcome measures, with a trend towards improvement in physical activity levels (physical activity in minutes per week at baseline was 1770 min, and 1909 min at six months, *p* = 0.13). Twenty volunteers, older adults, and staff were interviewed and found the intervention acceptable. The seated exercises were perceived as safe, manageable, and enjoyable.

**Conclusions:**

Trained volunteers can safely deliver online group exercise for community-dwelling older adults which was acceptable to older adults, volunteers, and club staff.

**Trials registration:**

NCT04672200.

## Introduction

Physical activity (PA) has multiple benefits for older adults, including reducing falls risk [1], improvement in physical function [2] and mental well-being [3]. Guidelines for older adults highlight the importance of engagement in daily PA, with a recommendation of achieving 150 min of moderate intensity aerobic activity per week plus strength and balance exercises twice a week [4]. Despite the clear benefits of PA, engagement in regular PA among community-dwelling older adults remains low [5], with common barriers including exertional discomfort, concerns with falling, and access difficulties [6].

There is increasing evidence on the role of trained volunteers in delivering interventions to promote increased PA among older adults. The Hospital Elder Life Programme [7] and the SoMoVe study [8] have shown that volunteers can be trained to engage with older inpatients to mobilise and perform exercises. We conducted a systematic review (8 studies) on the use of trained volunteers to deliver community-based PA intervention for older people and found that volunteer-led PA, including resistance exercise training, improved community-dwelling older adults’ functional status, frailty status, and reduced their fear of falls [9]. Notably, none of the included studies used online exercises.

The recent rise of the use of technology and the internet among older adults presents an opportunity to engage with older people online to promote increased PA. Digital technology has the potential of improving accessibility and promoting a wider engagement of older adults in physical activity interventions [10]. A recent scoping review, including 17 studies, showed variable adherence rates to online exercise among older adults ranging from 43 to 90% [11]. The current study explored the novel intervention of training volunteers to deliver online group exercise for older adults attending community social clubs. These are clubs based in the community where older people meet primarily for social reasons.

The specific aims of the study were:

1. To determine the feasibility of recruiting, training, and retaining volunteers to deliver online group exercises for older adults attending community social clubs
2. To explore the acceptability of the proposed intervention to older adults, volunteers, and club staff
3. To explore barriers and facilitators to the implementation of the intervention

## Methods

### Study design

This was a pre-post mixed methods study. Triangulation of quantitative and qualitative data was performed to explore the feasibility and acceptability of this intervention. Trials registration on ClinicalTrials.gov: NCT04672200 (17/12/2020).

### Volunteer recruitment and training

A detailed description of volunteer recruitment and training has been published in our protocol paper [12]. Volunteer inclusion criteria were adults aged ≥ 16 years, physically able to perform the exercises, and able to provide written consent. Key components of volunteer training included safe and effective delivery of the exercise intervention, techniques on how to lead an online group session and motivate participants, personal safety, participant safety, and documenting and reporting any adverse events.

### Participant recruitment

Older adults aged ≥ 65 years who attended community social clubs (*n* = 7) run by a charitable organisation in southern England were recruited by convenience sampling to the study between April 2021 and March 2022. Inclusion criteria included the ability to walk independently and provide written consent. Participants who were attending other exercise groups were excluded from the study as any improvement in outcome measures cannot be directly attributed to the volunteer-led intervention. A researcher visited the online social clubs to introduce the study. Participant information sheets and consent forms were posted to older adults who were interested in the study. Upon returning the consent forms, participants were invited to attend the online group exercise. A sample size of 30 participants was chosen in line with previous sample size recommendations for feasibility studies [13].

### Intervention

The intervention consisted of a once weekly volunteer-led online group exercise, for a duration of 30 min per session. The seated exercises focused on strengthening upper and lower limbs with the use of resistance bands and enhancing whole body range of motion and flexibility. Volunteers were trained to progress the exercises by encouraging participants to increase repetitions, increase the resistance by using a band with higher resistance and gently improve range of motion. For an in-depth description of the intervention, including how public contributors supported the development of this study including the intervention, please refer to our protocol paper [12]. Upon easing of COVID-19 lockdown restrictions in August 2021, some clubs returned to in-person visits, in which the intervention continued face-to-face.

### Data collection

Participants’ age, gender, marital status, care needs, cognitive function (Mini-mental state examination), co-morbidities (Charlson’s Co-morbidity index) and number of medications were recorded to provide participants’ baseline characteristics (Table 1). Data collection was conducted by a researcher at baseline and at 6 months.

**Table 1:** Club member participant characteristics

**Characteristics**	Baseline (*n* = 30)
Age^a^ (years)	77.3 ± 5.5
Gender	
Male	3 (10%)
Female	27 (90%)
Marital Status	
Divorced	2 (6.7%)
Married	9 (30%)
Single	4 (13.3%)
Widowed	15 (50%)
Care	
No Care	29 (96.7%)
Formal Provision	1 (3.3%)
Residence	
Private home living alone	16 (53.3%)
Private home living with other	10 (33.3%)
Sheltered accommodation	4 (13.3%)
T-MMSE^b^	25 (24–26)
Charlson Comorbidity Index^b^	4 (3–5)
No. of Medications^a^	4 ± 3
0–5	23 (76.7%)
6–10	6 (20%)
> 10	1 (3.3%)

*T-MMSE* telephone mini-mental state examination

^a^mean ± standard deviation; ^b^ median and interquartile range

### Primary outcome measures

The feasibility of implementing the intervention was determined by:

1. The number of volunteers recruited, trained, and retained
2. The number of older adults recruited
3. The number of physical activity sessions delivered, and proportion completed by participants (adherence)

The acceptability of the intervention, including barriers and facilitators, were determined through semi-structured interviews with older adults, their family members, volunteers, and club staff. Participants were selected by purposive sampling to ensure a wide range of views regarding the implementation of the intervention, including male and female participants, different clubs, and a representative age range. Interviews were conducted by SJM within the first 2 months and towards the end of the study (6 months) to ensure participants views were captured during the earlier stages of the intervention, and when the groups were well established. The interview schedules were underpinned by normalisation process theory (NPT) [14], providing a systematic approach to evaluate the implementation process. Interviews were conducted online, or by telephone, depending on participant preference. Telephone interviews were audio recorded with a Dictaphone, and online interviews were recorded using Microsoft Teams.

### Secondary outcome measures

The secondary outcome measures were:

1. Physical activity levels measured using the Community Health Model Activities Programme for Seniors (CHAMPS) questionnaire [15].
2. Activities of daily living measure using the modified Barthel Index [16].
3. Health-related quality of life (EQ-5D-5L) [17].
4. Sarcopenia measure using the Strength, Assistance with walking, Rising from chair, Climbing stars and Falls (SARC-F) questionnaire [18].
5. Frailty measure using the Program of Research to Integrate Services for the Maintenance of Autonomy (PRISMA-7) screening tool [19].
6. The cost of training a volunteer. This was estimated through calculating resource expenses for each volunteer, including provision of training booklets and exercise equipment, and costing the trainers time to conduct training, fidelity checks, and volunteer support meetings.

All outcome measures were recorded at baseline and repeated at 6 months. A descriptor for each of the outcome measures applied is available in our published protocol. Data collection was performed by a post-doctoral researcher (SJM) who is trained in administering these assessments.

#### Adverse events

Any injuries or symptoms developed directly as a result of the exercises were recorded as an adverse event. Volunteers were trained to document any adverse events. All adverse events were reported to the research team. The clubs also have established procedures for responding to incidents and accidents including access to emergency contacts of participants.

### Analysis

Baseline characteristics of participants were reported as mean (SD) or median (interquartile range (IQR)) for continuous variables and number (percentage) for categorical variables. Descriptive statistics were used to analyse the number of volunteers and participants recruited, the number of volunteers trained and retained, and participants’ adherence to the intervention. Secondary outcome measures recorded at baseline were compared at 6 months using t-tests or Wilcoxon signed rank tests depending on the normal distribution of data. Statistical significance was considered when *p* < 0.05. Analyses were conducted using statistical software SPSS (Version 25).

Interviews were transcribed verbatim and analysed using reflexive thematic analysis [20] by SJM. This approach included: phase 1—familiarising with the data, phase 2—generating initial codes, phase 3—searching for themes, phase 4—reviewing themes, phase 5—defining and naming themes and phase 6—producing the report. The analysis was guided by NPT and conducted using NVivo (version 12). Codes were interpreted to determine the acceptability of the intervention, including barriers and facilitators of the implementation process, and then organised into themes that reflected the views of participants regarding the online exercise intervention.

### Ethics

This study received ethical approval from the University of Southampton Faculty of Medicine Ethics Committee and Research Integrity and Governance committee (ID: 52 967.A1). The study steering committee had oversight of study processes and research personnel were trained in Good Clinical Practice. Data was anonymised and stored on a password protected University database and handled in line with the Data Protection Act 2018 to maintain confidentiality.

## Results

### Feasibility of the volunteer training programme

Nineteen volunteers were recruited, 15 completed training and 9 volunteers (47.3%) were retained at the end of the study. Four volunteers withdrew before training due to beliefs that they were unsuitable for the role, including lack of time to commit, and poor confidence in their skills to deliver exercise. Six volunteers withdrew after training because of: ill health (1), work commitments (1), concerns regarding the safety of online exercise (2), and reduced commitment to the volunteer role with the return of normal activities after the Coronavirus pandemic 2019 (COVID-19) lockdown (2).

All volunteers completed 3 online group training sessions, 60–90 min, with 1 trainer (SJM), and additional online one-to-one training depending on competence and confidence (33% of volunteers). In total, each volunteer completed a median of 2.7 h (interquartile range [IQR] 2.7–3.3) of training. The trainer (SJM) was a qualified and experienced exercise practitioner. Volunteers were mainly female (78%), mean age 68 (± 6.3) years (age range 59 – 77 years), retired (67%), with previous volunteering experience (78%), but no experience delivering exercise (78%). The trainer (SJM) visited clubs to support volunteers, conducted fidelity checks, and organised 5 volunteer group support meetings.

### Feasibility of delivering the exercise intervention

Seven community social clubs, comprising 62 members, were approached through online visits from the study team and encouragement from club staff. Thirty-four older adults were recruited, and 30 were retained at the end of the study. Reasons for withdrawal were unrelated injury or ill health (3), and one participant stopped attending the club. Participants were mainly female (90%) living at home without formal care (Table 1).

Overall, volunteers delivered 184 group weekly exercise sessions (127 online; 57 in-person) March 2021 to April 2022. There was considerable variability in the number of sessions delivered per volunteer (range 11–67; median 35.0 [IQR 20.0–37.0]) related to time of enrolment, availability of volunteer time, and number of clubs led per volunteer. Over a 6-month data collection period, comprising 1 volunteer-led session per week, participants’ attendance ranged from 4.17–100% (median 54.17% [IQR 37.5–77.1]). Twenty-six participants completed the intervention online (median 54.17% [IQR 42.71–66.67]), 1 participant transferred from online to in-person post lockdown, and 3 participants attended only in-person sessions (median 83.33% [IQR 60.42–85.42]).

### Secondary outcome measures

Light PA increased 90 min per week (*p* = 0.08, *d* = 0.33), and individuals meeting recommended PA levels improved from 33% at baseline to 43% at 6 months (Table 2). There were no changes in quality of life, frailty, or sarcopenia risk (73% classified as not frail and at low risk of sarcopenia). Two minor adverse events were reported by two participants, one involving an exacerbation of arthritic knee pain and another an exacerbation of previous injury. The cost of training a volunteer was estimated to be £220.75.

**Table 2:** Secondary outcome data

**Outcomes** Median (Interquartile Range)	Baseline (*n* = 30)	Post 6-months (*n* = 30)	Significance (*P* < 0.05)
PA Time (min) / Week			
Light	1530 (1286.25–1893.75)	1620 (1492.50–2002.50)	*p* = .08
Moderate	105 (0–258.75)	105 (22.50–255.00)	*p* = .27
Vigorous	0 (0–0)	0 (0–0)	*p* = .89
Moderate-Vigorous	105 (0–258.75)	105 (22.50–255.00)	*p* = .39
Total	1770 (1391.25–2062.50)	1905 (1537.50–2407.50)	*p* = .13
PA Category			
Low (< 150 min Mod-Vig/Wk)	20 (66.7%)	17 (56.7%)	
Medium (≥ 150 min Mod-Vig/Wk)	10 (33.3%)	13 (43.3%)	
Modified Barthel Index	19.0 (18.8–20.0)	19.0 (18.8–20.0)	*p* = .77
	15.0–20.0 (range)	15.0–20.0 (range)	
EuroQol State			
Mobility	1.5 (1.0–3.0)	1.5 (1.0–2.3)	*p* = .21
Self-Care	1.0 (1.0–1.0)	1.0 (1.0–1.0)	*p* = 1.0
Usual Activities	1.0 (1.0–1.3)	1.0 (1.0–2.0)	*p* = .08
Pain/Discomfort	2.0 (1.0–3.0)	2.0 (2.0–3.0)	*p* = .05
Anxiety/Depression	1.0 (1.0–2.0)	2.0 (1.0–2.0)	*p* = .11
EuroQol VAS	77.5 (63.8–80.0)	77.5 (67.5–85.0)	*p* = .64
EuroQol Index	0.859 (0.798–0.926)	0.846 (0.712–0.901)	*p* = .05
Sarcopenia SARC-F	1.0 (.0–4.0)	1.0 (.0–3.3)	*p* = .26
Low Risk	22 (73.3%)	23 (76.7%)	
High Risk	8 (26.7%)	7 (23.3%)	
Frailty PRISMA-7	2.0 (1.0–3.0)	2.0 (1.0–3.0)	*p* = .5
Not Frail	22 (73.3%)	22 (73.3%)	
Frail	8 (26.7%)	8 (26.7%)	

Metabolic equivalent; *PA* physical activity, *VAS* visual analogue scale

### Acceptability of the intervention

**Fig. 1:**
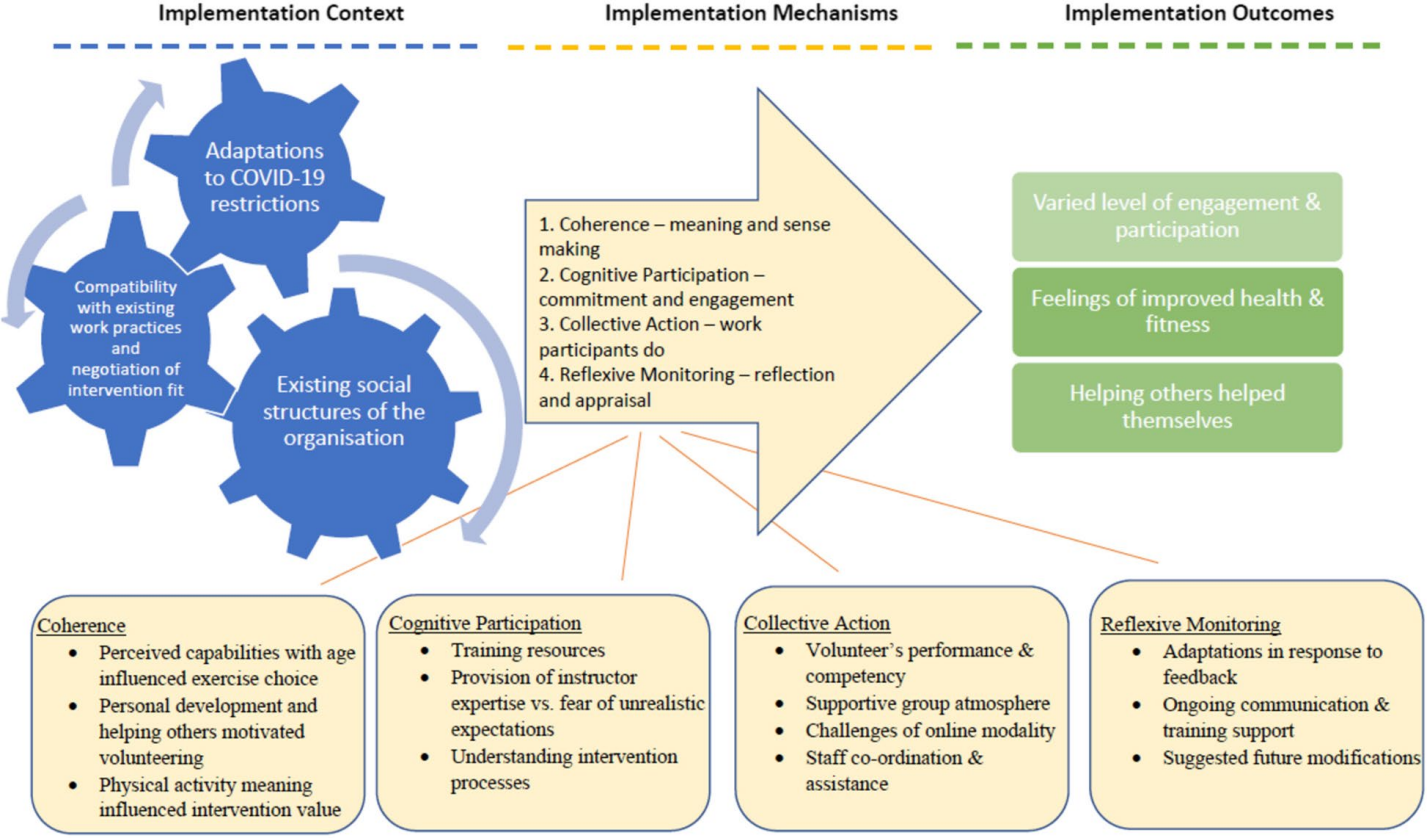
Acceptability of the intervention

Seven volunteers (aged 57–83 years; 6 female), eight older adults participating in the exercise intervention (aged 68–82 years; 8 female), one family member (aged 67 years; female), and four staff members were interviewed. Results are presented under the main domains of NPT, including implementation contexts, mechanisms, and outcomes (Fig. 1). Quotations supporting each theme are showcased in Table 3.

**Table 3:** The main themes and subthemes influencing the acceptability of the intervention, including quotations

**Main theme: Implementation Context**		
**Subtheme L2 (No. of codes)**	**Subtheme L1 (No. of codes)**	**Quote Examples**
Adaptations to COVID-19 restrictions (146)	Fear of infection (20)	‘Some of them (older adults) have deteriorated. Because people have lost confidence in everything… some people are just scared to go out again.’ (Volunteer)
	Changed PA opportunities (63)	‘I couldn’t go to my Zumba class which I love, which I still can’t go to at the moment which is annoying. So, as soon as they mentioned the exercise classes, I jumped at the chance.’ (Participant)
	Altered club functioning (63)	‘We started providing a lot of support to people in their own homes during COVID; telephone support, the online clubs, and bits of digital connection work… so we've got more to offer older people that become members.’ (Staff)
Existing social structures of the organisation (142)	Ethos and objectives (89)	‘For me …. it’s about combating isolation in our elderly community. People who are alone, or even as couples, it’s about getting out and about and continuing to show interest and motivation in different activities… that’s why we offer such a wide choice of activities so there’s something for everyone.’ (Staff)
	Characteristics of clubs (53)	‘I notice with the people that I’m with at (the club), most of them are elderly widows. They are all very good at joining in with activities and I’m sure that is due to the fact that they are alone.’ (Participant)
Compatibility with existing work practices and negotiation of intervention fit (76)	Club resources (45)	‘I mean that is the only way we can do it (offer exercise), to be honest, cos obviously there’s not enough staff and we haven’t got the funds to pay people. And I think it’s good to utilise their (volunteers’) skills.’ (Staff)
	Normal club routine (31)	‘It seems like a good set of exercises that people are willing to do and enjoy within that sort of time frame. We then spend the rest of the session doing something else, you know, it (exercise) is part of the session, not the whole thing.’ (Staff)
**Main theme: Implementation Mechanisms**		
**Subtheme L2 (No. of codes)**	**Subtheme L1 (No. of codes)**	**Quote Examples**
Coherence (844)	The intervention suited changes in perceived capabilities with age, but for those more able the intervention was not challenging enough (202)	‘I suppose the fact that it's seated, and other people feel a lot more comfortable with that, they feel they're not going to be asked to do press ups or jump up and down or anything… But the fact that it's seated, the fact that it is relatively easy and the fact that you can stop if you feel uncomfortable, I think helps’. (Volunteer) ‘I think the exercises are for people who are older, like more 75 to 80 plus. I think it would be nice if we could have exercises for the newly retired 60 plus to 75, because they are fitter, and they are losing their mobility. So, it’s finding the middle ground. What we’re doing at the moment is very much helping the less abled, but we need something a bit more lively.’ (Staff)
	PA meaning influenced perceived value of the intervention (612)	‘I used to go to a gym with a lot of young people, you feel a bit threatened when you go into that environment. Because you know that you are flabby and not as fit as some of them, and some people in the gyms that I've been to you think, “goodness me what are they gonna think of me?” So, if you've not exercised for a long time or not exercise regularly, you can feel a bit put off by the culture of “keep fit”.’ (Volunteer) ‘I think if you’re not active you seize up a bit, the joints seize up. I mean, I’ve got a bit of arthritis in my knee, and I find it better when I’m exercising more. It gives out on me now and again, but if I keep exercising regularly it doesn’t seem to happen, and it’s not painful.’ (Participant)
	Personal development and helping others motivated volunteering (30)	‘I can help them (exercise participants)… if it (exercise) helped me, it can certainly help them and I think it is very important to keep some sort of fitness in your schedule….. So, I want to share my experience, and what good it had done, with others.’ (Volunteer)
Cognitive participation (162)	Level of understanding in intervention processes influenced engagement (35)	‘I think everyone remembers Dr (name) coming in to chat and he explained about the programme and asked who would be willing to train. Several people put their hands up, so I think that’s a very good way. It’s somebody sort of coming from the University and explaining what it’s all about.’ (Volunteer)
	Training resources impacted intervention functioning (87)	‘I’ve got all the training, all the bits and pieces, all the literature, it was well designed, well written and well explained. I thought the video was very good, and I thought you (trainer) were very good.’ (Volunteer)
	Tensions between provision of instructor expertise and fear of unrealistic expectations (40)	‘I think an external instructor would be better because they know, I mean after all, (name) is just somebody who volunteered out of the goodness of her heart, to do the best she can. She does it as well as she can, but that’s not the same as somebody who knows about the exercises.’ (Member) ‘Someone coming in (instructor) might have expectations about us that we are not sure about’ (Member)
Collective action (599)	Volunteer’s performance and competency delivering exercise (285)	‘I feel as if I’m an amateur delivering them (exercise). I feel as if I’m doing my best, I’m doing the exercises and people are following along, but I don’t feel 100% competent because I can’t remember all the exercises, and which muscles they’re exercising.’ (Volunteer) ‘I think we feel they (volunteers) are just one of us; they’re not an outsider coming in and therefore we’re relaxed with them… we are already friends, we are not self-conscious when they are around’ (Member)
	Co-ordination and assistance provided by staff (39)	‘She (staff member) just welcomed me so much. Because I struggled at the beginning, but (name), she was the right person for me, for me to go to first.’ (Participant)
	The supportive group atmosphere aided implementation (108)	‘You spend a lot of time at home on your own, and I think you need things to look forward to, and to go to something like that (online exercise group), it is important to be sociable with people… I’ve really enjoyed doing it because it keeps you in touch with people, especially during this COVID.’ (Member)
	The online modality presented new challenges to overcome (167)	‘I think that Zoom has been very limited; there’s only about 10 of us. And several others I meet when I go to the shops, I say, “are you not going to come on Zoom?” And they say, “no we will wait until the clubs come back again”. It may be practicalities of technology and things like that… We are not all computer savvy,’ (Member)
Reflexive monitoring (146)	Ongoing communication and training support (56)	‘I think the volunteers feel valued by the fact you come along and keep encouraging and advising them as to what they’re doing. And I think that is immensely valuable to them.’ (Staff)
	Adaptations to the intervention in response to feedback (34)	‘With the bands now, it’s a new toy to play with. Otherwise, if it’s the same old thing, week in, week out, it gets a bit boring. You gotta challenge yourself or make it slightly different, put a different perspective on it.’ (Volunteer)
	Suggested future modifications (56)	‘So I think the idea of changing the routine would be a good help because we've been through the set (exercises) several times.. You’ve gotta do your warm-up and your warm down, but something different in between might be a lot nicer for them (participants). I think a bit of variety would be nice.’ (Volunteer)
**Main theme: Implementation Outcomes**		
**Subtheme L1 (No. of codes)**	**Quote Examples**	
Varied level of engagement and participation (59)	‘I don’t do them (exercises) every day, but I’ve got the booklet. And then I might think, “cor, I’ll just do them while I’m sat here”. You can do it while you’re watching tele if you want… It’s quite good to be able to do while I watch Wimbledon.’ (Member)	
Feelings of improved health and fitness (121)	‘I find my body is more flexible. Because I know when I reach up in my kitchen cupboards, I can do it easier now I’ve been doing the exercises.’ (Member) ‘Seeing people online, now that we are doing Zoom, makes you feel, “hey that’s great there’s another person here!” We can talk.. see other people… I do think that seeing other people is a very important part of well-being.’ (Volunteer)	
Helping others helped themselves (66)	‘I feel like I've done something, I've helped… but that's why I do the volunteering because I feel like I'm of some use. It's some reason to get up in the mornings, and some reason to do something… I'm enjoying myself, I'm being a human.’ (Volunteer)	

### Implementation contexts

#### Adaptations to COVID-19 restrictions

Government restrictions during the pandemic reduced participants’ social interactions and created a sense of isolation. Fear of infection altered daily activity choices and behaviours, such as reduced confidence to leave the house. Subsequently, many participants experienced reductions in their normal PA routine and the pandemic accelerated organisational change at the clubs, including expansion of club activities, and provision of online options and remote support to club members. The intervention created an opportunity to exercise during social isolation and replaced participants’ normal PA routines.

#### The existing social structures of the organisation

The characteristics of the social clubs impacted the reach of the intervention. For instance, most participants were widowed women and there was a lack of male older adults, impacting diversity. The organisation’s ethos and objectives, to enhance members’ mental health and well-being was consistent with the underlying principles of the intervention, improving acceptability from key stakeholders, including club managers and staff.

#### Compatibility with existing work practices and negotiation of intervention fit

Incorporation of exercise challenged some of the participants’ normal sedentary routines and preferred activities, such as the expectation of attending the club to have ‘a chat with friends and listen to a talk’ (Volunteer). To prevent interference with other social activities a shorter exercise duration was introduced (30 min).

The organisation’s budget restricted funds for a professional exercise instructor, therefore, the volunteer-led intervention was compatible with the organisation’s working practice, in which, ‘the aim is for the Clubs to be run by volunteers, with staff support.’ (Staff). Subsequently, volunteer-led exercise was implemented on a regular basis compared to expensive one-off implementation of external instructor support.

### Implementation mechanisms

#### Coherence

##### The intervention suited changes in perceived capabilities with age, but for those more able the intervention was not challenging enough

Reduced functional capacity with age altered perceived capabilities for PA and increased perceptions of harm, including participants’ worry regarding over exertion, discomfort, and injury. Older adults who felt vulnerable and unable to cope with exercise were embarrassed showing this vulnerability to others during activity. The chair exercise suited lower fitness levels and individuals who were unfamiliar or felt more vulnerable during PA. Participants described the exercise as comfortable and safe, ‘they’re quite good exercises because they’re not over taxing’ (Member). Fitter participants found the exercises too easy and would have preferred more challenging and energetic forms of exercise. Staff perceived the intervention as more appropriate for their oldest old members and thought their newly retired members would prefer more challenging and ‘lively’ exercise.

##### PA meaning influenced the perceived value of the intervention

Each participant had their own sense of meaning and value attached to PA, which influenced intervention coherence. PA meaning was influenced by a range of factors, including PA knowledge, familiarity and history of PA, the opportunities afforded by the environment, and wider social culture. For instance, some older adults had perceptions that they did not belong in a traditional ‘keep fit’ culture, where they often felt ‘threatened’ in a gym environment.

Most participants recognised the benefits of PA and were motivated to participate in the intervention to improve fitness, manage chronic health conditions, prevent deterioration with ageing, improve well-being, and enhance functional ability and activities of daily living. For instance, a participant described PA as important in maintaining her joint health and improving self-esteem.

##### Personal development and helping others motivated volunteering

Volunteers were enthusiastic to support member’s health and well-being and were motivated to volunteer to overcome feelings of loneliness, combat negative body image stereotypes and have a sense of purpose through contributing specific skills at the club.

#### Cognitive participation

##### Level of understanding in intervention processes influenced engagement

Overall, a good rationale to the project was provided. Volunteer recruitment was influenced by the clarity of the volunteer role and the training received. Club visits from health professionals piqued participant interest and understanding.

##### Training resources impacted intervention functioning

Training content was valued by volunteers, ‘I think the training, the information, and the support we’ve been given has been really good.’ (Volunteer). Volunteers’ also felt the training enhanced their confidence and competence to deliver the exercise. The booklets and videos provided a resource to practice at home and most volunteers referred to the booklets to prompt them during the sessions.

##### Tensions between provision of instructor expertise and fear of unrealistic expectations

Perceptions regarding professional exercise instructors influenced participants’ commitment and engagement with the volunteer-led intervention. Some participants preferred the expertise of a qualified instructor, especially those who wanted specialist support for health conditions. However, participants also expressed feeling self-conscious exercising with qualified instructors and perceived that they may have unrealistic exercise expectations through a lack of empathy for older people, particularly in the capabilities of older people to perform certain exercises or achieve certain goals. Comparatively, the exercise volunteers at the social clubs were a similar age and ability, which enhanced participant motivation and confidence (detailed in the *collective action* theme below).

#### Collective action

##### Volunteers’ performance and competency delivering exercise

The volunteers demonstrated competency when delivering the exercise intervention including guiding participants’ exercise technique, learning pacing, effectively setting up devices for online demonstrations, and implementing safety considerations. Members seemed satisfied with the volunteer’s exercise delivery, ‘I think (name) did a really good job, she got the hang of it, and got us all doing them properly so, it was good. I don’t think we needed a professional.’ (Member).

Nevertheless, they were not qualified instructors and there was a limit to their knowledge and to what they could deliver. One volunteer was concerned about her competence to meet vulnerable older adult’s needs and described how she felt like an ‘amateur’.

The members regarded the volunteers as positive and relatable role models, due to their similar age and abilities. The volunteers had a strong rapport with the group, which helped to create a fun atmosphere in which the exercise was delivered in a relaxed and non-judgmental way. Learning from peers created a positive vicarious experience for participants, bolstering their confidence and engagement with the exercise routine. Volunteers brought their own skill sets and style to the exercise role. Two volunteers sharing the role facilitated delivery through reducing pressure on volunteers and bolstering confidence through peer support.

##### Co-ordination and assistance provided by staff

Staff were key in the smooth running of the intervention. They effectively organised the social clubs, including helping with volunteer recruitment, integrating new volunteers into the social groups, and facilitating club safety. Effective communication between staff and trainers was essential to provide a bridge between trainers and volunteers.

##### The supportive group atmosphere aided implementation

Exercising in a group helped reduce isolation and motivated engagement with the intervention. Social connections were important to participants, in which the group created a sense of belonging, moral support and a shared experience.

##### The online modality presented new challenges to overcome

Older adults familiar with online technology were more likely to engage with the intervention compared to individuals with a fear and lack of knowledge. The clubs had lower numbers attending the online groups, which impacted the reach of the intervention. Nevertheless, the organisation loaned devices to improve member access and they provided learning and support (digital coaching) to resolve any technical issues, helping older adults to upskill, as illustrated by this staff member:

‘Her face when she finally could see people… It did take us several days, a lot of hours on the phone, but when she suddenly popped up on that screen and she realised she’d done it, it was just amazing. So, it’s pushed people so far out of their comfort zone.’ (Staff).

Inevitably there were technical difficulties experienced during online exercise, such as difficulties connecting, and poor sound, or picture quality. Staff assisted participants online, putting volunteers on ‘spotlight’ to improve visibility of demonstrations and setting up the online meetings. Family members also supported older adults with technology use. However, the volunteers felt that delivering the exercise online compromised interaction and coaching due to the inability to clearly see the group (e.g., small screens, poor set up of camera positioning). This created some safety concerns. To reduce injury risk and to follow the organisation’s insurance policy, the exercises were completed seated. However, strict safety guidance limited the effectiveness of exercise for individuals who required more challenging standing movements, particularly participants who wanted to improve balance.

#### Reflexive monitoring

##### Ongoing communication and training support

To encourage and support volunteers, trainers provided regular communication in the form of phone calls, emails, and club visits, which made volunteers feel ‘valued’ and gave the opportunity for feedback and continued learning. Regular volunteer meetings allowed shared experiences with peer feedback and support. Moreover, fidelity checks conducted by the research team facilitated volunteer development:

‘It was good to have like the one to one, sort of examination …somebody watching you to make sure that you knew what you were doing before you started.’ (Volunteer).

##### Adaptations to the intervention in response to feedback

Listening and responding to feedback from regular communication with volunteers, participants, and staff was essential to improve the acceptability of the intervention. Feedback was appraised and resulted in several changes to the intervention. Additional warm up and cool down exercises were added, and resistance bands were introduced to improve strength and interest and to progress the exercises.

##### Suggested future modifications

Participants suggested exercise variety could keep the intervention ‘fresh’ and enhance enjoyment. Some volunteers disliked the label ‘exercise volunteer’ due to negative fitness stereotypes and preferred an emphasis on ‘mobility’, which they thought would attract more volunteers to the role. Volunteer recruitment was essential for intervention functioning, which participants suggested could be improved by providing an exercise taster, inclusion of a monthly newsletter, and hosting a volunteer event to show appreciation and thanks. To enable embedding of the intervention and provision of support moving forward, staff wanted ongoing links with the University.

#### Implementation outcomes

##### Varied level of engagement and participation in the exercise

Engagement in the intervention varied. While most members were keen to participate, some felt indifferent, or preferred not to exercise. Overall, the intervention became an integrated routine at the start of the club, and some members completed the exercise in their own time. Staff commented that inclusion of the intervention could help retain and attract new members and volunteers to the organisation:

‘I think for recruitment, actually saying “if you’re interested in exercise training, we could provide that”. It’s a hook for members so it would probably be a hook for volunteers as well.’ (Staff).

##### Feelings of improved health and fitness

The exercises introduced new types of movement and participants described improved strength, posture, balance, mobility, and flexibility. Moreover, the social connections from the group exercise enhanced well-being and improved participants’ mood.

##### Helping others helped themselves

The exercise role provided volunteers with a sense of belonging which helped improve well-being. Volunteering enhanced personal growth and development, such as increasing leadership skills. The role gave volunteers a sense of purpose and self-esteem, as well as increasing their own PA levels.

## Discussion

It was feasible and safe to deliver a volunteer-led online exercise intervention in social clubs for community-dwelling older adults. The intervention was acceptable to staff, volunteers, and older adults. Volunteers were positive and relatable role models who developed a non-judgmental group atmosphere. The group environment, social connections, and sense of togetherness motivated participation. A key to success was the digital coaching and support to upskill older adults’ technological knowledge and improve access and confidence to engage with the intervention online. Volunteer retention rate was at 47.3% at the end of the study period which is in line with the usual retention rates for volunteer-based interventions [8, 21].

This study adds to a burgeoning evidence-base suggesting that with proper training, volunteers can take on more direct roles in supporting older adults’ PA [8, 22] and can successfully deliver an exercise intervention to community-dwelling older adults online [10, 11]. A systematic review of 12 studies found evidence suggesting that volunteer-led PA interventions that include resistance exercise training, can improve outcomes of community-dwelling older adults including functional status, frailty status and reduction in fear of falls [9]. This study adds to existing research through exploring how best to recruit, train, and retain volunteers to deliver PA interventions, and in an online mode. The training programme adequately prepared volunteers for the role. Videos and booklets provided volunteers with learning tools to practice from home. Several factors influenced volunteer engagement and retention, including ongoing support and communication from trainers and staff, flexibility to adapt the intervention in response to feedback, and volunteers’ desire for personal development, helping others, and establishing a specific role and contribution to the club. Regular group volunteer meetings allowed peers to share experiences and feedback and provided a valued support mechanism.

This feasibility study demonstrated encouraging trends in PA levels and subjective reports regarding enhanced health and fitness, but these preliminary findings need to be confirmed in a larger controlled trial. Importantly, the intervention provided opportunity for participants to exercise during the pandemic, a time when older adults experienced significant restrictions to activities of daily living with subsequent deconditioning and reductions in health-related quality of life [23, 24]. The intervention also improved volunteers’ PA opportunities and volunteering enhanced volunteers’ personal growth and development, gave them a sense of purpose, enhanced their self-esteem, and improved well-being. This is consistent with previous research that showed volunteering in later life was associated with improved welfare, enhanced life satisfaction, alleviated loneliness, and was an important source of social capital [25–27]. Encouraging volunteering in later life is an important health and social care strategy, and this study has helped to develop training resources and support mechanisms that can be utilised in practice to improve the accessibility of volunteering for older adults.

Most volunteers were aged over 60 years with similar ability levels to the groups they were leading, and some of them also experienced their own health problems. As such the volunteers became relatable role models in which they provided a positive vicarious experience for club members, increasing their confidence (i.e., self-efficacy to exercise) and encouraging them to join in the exercise sessions. Exercise self-efficacy refers to the participants’ beliefs in their capabilities to exercise, and can influence choice to participate, level of effort exerted, and perseverance in the face of difficulties [28]. Strong normative effects through exercising with peers (i.e., vicarious experiences) have shown significant associations with older adults’ perceived self-efficacy and predicted PA levels in previous research [29]. Considering the presence of negative ageing stereotypes and exercise misconceptions, such as feeling out of place in society’s ‘fitness culture’, watching similar others (volunteers) engage successfully in exercise (i.e., positive vicarious experiences) was important to increase older adults’ beliefs in their own capabilities to be active and encourage participation in the current intervention. Future research is needed to explore these links further, investigating the impact of volunteer-led exercise on self-perceptions of ageing and PA [30]. Moreover, considering some of the misconceptions older adults had surrounding ‘exercise’, care should be taken when labelling volunteer roles in future interventions, perhaps emphasising ‘mobility’ and ‘health’ to optimise volunteer recruitment [31].

A key influence on the success of the intervention was participants’ digital literacy and their ability to access the online exercise. Older adults are more likely to experience barriers in the use of digital tools, such as poor prior experience with technology, and cybersecurity concerns [32, 33]. The Survey of Health, Ageing and Retirement in Europe (SHARE) found that 49% of older adults aged ≥ 50 years used the internet [34]. The pandemic has prompted older adults to adopt new technologies to facilitate their tasks (e.g., online shopping) and to guard against loneliness and social isolation through learning how to use applications for virtual meetings and communication [35]. Digital educational strategies, such as the Digital Literacy Workshop for the Elderly, demonstrated the feasibility and acceptability of training older adults to obtain digital skills during the pandemic [36, 37]. Likewise, this study has shown that it is feasible to deliver an online volunteer-led exercise intervention to community-dwelling older adults through supporting them to access, learn and develop skills in digital literacy. Specifically, the host organisation provided devices, online platform educational workshops, and unlimited phone support to participants.

A motivating factor to learn how to use the online platform was to continue socialising, and the sense of belonging and trust established in the social groups. Hence, the group nature of the intervention was important. Similarly, a range of studies have pinpointed group exercise and social connection as a strong motivator to exercise in later life and a principal influence on exercise adherence [38–41]. While face-to-face exercise was preferred by volunteers and older adults for meeting and exercising, the online intervention was the next best thing during activity restriction and confinement in the pandemic era. Future interventions should consider barriers to online exercise, such as volunteers’ difficulty observing and therefore coaching participants online, and facilitators, such as effective set-up of devices, using ‘spotlight’ functions to improve visibility of exercise demonstrations, and establishing a supportive group atmosphere. Upon easing of COVID-19 restrictions some social groups returned in-person and continued the exercise intervention in a face-to-face setting. Most participants completed the intervention online during the 6-month data collection period, only 3 participants started the intervention at in-person clubs. Future research should consider comparing older adults’ adherence rates and health outcomes between online and in-person PA interventions.

Robust collaborations with the host organisation, including valuable input from staff, volunteers and participants was a strength of this study. A mixed methods approach allowed rich in-depth qualitative exploration and understanding of implementation processes, while quantitative measures revealed the impact of the intervention on physical activity and health outcomes. A further strength was the use of NPT, which provided a set of conceptual tools to support understanding and evaluation of the adoption, implementation, and sustainment of the intervention, and considered the complexity of the beliefs, behaviours, artefacts, and practices that played out over time and between settings [14, 42, 43]. Although collaboration was key to the success of the study, results were limited to a small section of society (i.e., older adults attending social clubs), in which most participants were white widowed women. Future research should explore the feasibility of volunteer-led online exercise interventions within a wider diversity of older adults, including more men and a stronger representation of multiple ethnic groups, and within different community settings, such as care homes.

### Limitations

The study was conducted during the COVID-19 pandemic and changes in social distancing rules resulted in a change of how the intervention was delivered towards the end of the trial period. Club members were very keen to return to face-to-face meeting following a prolonged period of social restrictions and therefore the intervention was moved from online to face to face for 2 clubs towards the end of the intervention. Meeting face-to-face may have introduced bias to the study as it may have enhanced its effects but as no changes were made to the exercises including the duration and frequency of the intervention, the effects are likely to me minimal. This study does not have a control group but as the main aim of the study was to determine the feasibility and acceptability of the intervention, key findings to be explored in this study could be achieved without having a controlled group. Another limitation of this study was the low adherence rates to the online exercise intervention at 54%. However, this appears to correlate with existing online physical activity intervention adherence rates which ranges from 43–90% [11]. Further research is needed to better understand factors that influence participants’ adherence to online exercise intervention and strategies to improve this.

## Conclusions

This study demonstrated that it was feasible and safe to deliver a volunteer-led online exercise intervention in social clubs for community-dwelling older adults, including recruitment, training, and retention of volunteers. The intervention was acceptable to staff, volunteers, and club members. Supporting participants digital literacy skills and promoting a positive group exercise environment were key to the success of the intervention. A future controlled trial is needed to explore the impact of volunteer-led exercise interventions on community-dwelling older adults’ health outcomes and across community settings.

## Data Availability

The datasets used and/or analysed during the current study are available in the PURE repository of the University of Southampton and will be made available from the corresponding author on reasonable request.
